# Diving into the Evolutionary History of HSC70-Linked Selective Autophagy Pathways: Endosomal Microautophagy and Chaperone-Mediated Autophagy

**DOI:** 10.3390/cells11121945

**Published:** 2022-06-16

**Authors:** Simon Schnebert, Maxime Goguet, Emilio J. Vélez, Alexandra Depincé, Florian Beaumatin, Amaury Herpin, Iban Seiliez

**Affiliations:** 1 E2S UPPA, INRAE, NUMEA, Université de Pau et des Pays de l’Adour, 64310 Saint-Pée-sur-Nivelle, France; simon.schnebert@inrae.fr (S.S.); maxime.goguet@inrae.fr (M.G.); emilio-jose.velez-velazquez@inrae.fr (E.J.V.); florian.beaumatin@inrae.fr (F.B.); 2 UR1037 Laboratory of Fish Physiology and Genomics, Campus de Beaulieu, INRAE, F-35042 Rennes, France; alexandra.depince@inrae.fr

**Keywords:** chaperone-mediated autophagy, CMA, LAMP2A, HSC70, KFERQ, endosomal microautophagy, eMI, evolution

## Abstract

Autophagy is a pleiotropic and evolutionarily conserved process in eukaryotes that encompasses different types of mechanisms by which cells deliver cytoplasmic constituents to the lysosome for degradation. Interestingly, in mammals, two different and specialized autophagic pathways, (i) the chaperone-mediated autophagy (CMA) and (ii) the endosomal microautophagy (eMI), both rely on the use of the same cytosolic chaperone HSPA8 (also known as HSC70) for targeting specific substrates to the lysosome. However, this is not true for all organisms, and differences exist between species with respect to the coexistence of these two autophagic routes. In this paper, we present an in-depth analysis of the evolutionary history of the main components of CMA and eMI and discuss how the observed discrepancies between species may contribute to improving our knowledge of these two functions and their interplays.

## 1. Introduction

**Figure 1:**
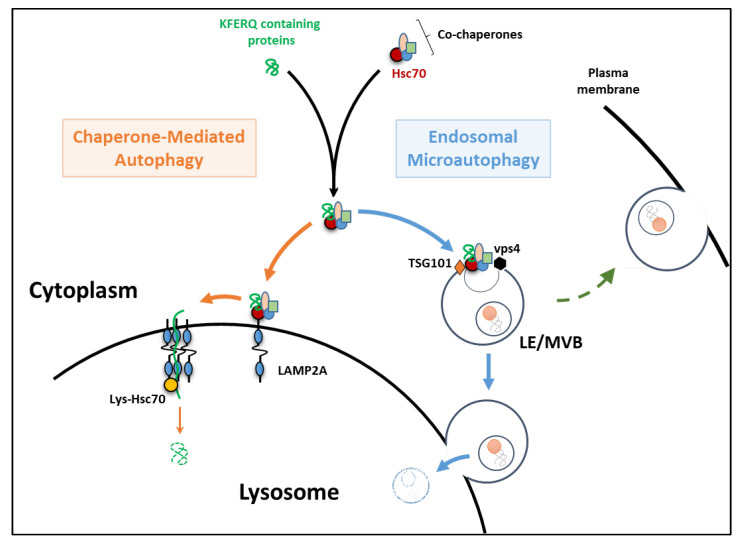
Chaperone-mediated autophagy (CMA) and endosomal microautophagy (eMI) as described in mammals. The first step (dark arrows) of recognition of the KFERQ motif-containing proteins by HSC70 and its co-chaperones is shared by both pathways. During CMA (orange arrows), the substrate-chaperone complex is then directed to the lysosomal membrane through specific binding to the cytosolic tail of the lysosomal-associated membrane protein 2A (LAMP2A). LAMP2A then organizes into a multimeric complex that allows substrates to translocate across the lysosomal membrane and reach the lumen for degradation by lysosomal hydrolases. During eMI (blue arrows), the substrate-chaperone complex is transported to late endosomes/multivesicular bodies (LE/MVB) by direct binding of HSC70 to phosphatidylserine residues on the LE/MVB membrane. Members of the ESCRT machinery (including VPS4 and TSG101) will then mediate the internalization of the substrate into intraluminal vesicles. eMI substrates may then be degraded within the LE/MVB itself or upon their fusion with a lysosome. Alternatively, eMI could mediate extracellular protein releases due to the ability of LE/MVB to fuse with the plasma membrane (green dashed arrow).

In the late 80s, Fred J Dice’s work was pioneered when it first described a lysosomal pathway selectively degrading soluble proteins [1]. At that time, selectivity in lysosomal degradation had not yet been established or even considered. This notion has since gained ground, and the mechanism that Fred J Dice discovered is nowadays known as chaperone-mediated autophagy (CMA, Figure 1). In detail, during this process, cytosolic proteins containing a pentapeptide sequence sharing biochemical similarity to KFERQ (lysine-phenylalanine-glutamate-arginine-glutamine) are first recognized by the heat-shock protein family A [Hsp70] member 8 (HSPA8/HSC70) and co-chaperones (including Hsp90, Hsp40, the Hsp70-Hsp90 organizing protein (Hop), the Hsp70-interacting protein (Hip), and the Bcl2-associated athanogene 1 protein (BAG-1)) [2,3]. The substrate-chaperone(s) complex then docks at the lysosomal membrane through a specific binding to the cytosolic tail of the lysosomal-associated membrane protein 2A (LAMP2A), which is considered to be the rate-limiting and essential protein for CMA [4]. LAMP2A then organizes into a multimeric complex that allows substrates to translocate across the lysosomal membrane [5,6], where they are ultimately degraded by acid hydrolases. Beyond these primary modalities, in recent years, many research efforts have been carried out to decipher the ins and outs of how the pleiotropic physiological function(s) of CMA exert and are regulated to obtain specificity [7]. Hence, recent findings emphasized the fundamental role(s) of CMA in regulating numerous cellular functions, including cellular energetics, transcriptional programs, cell death, cell survival mechanisms, and even DNA repair, whose main players harbor one or more KFERQ domains.

Very recently, along with the emergence of new concepts in the field of autophagy, Sahu et al. evidenced that KFERQ domain-containing proteins can additionally be subjected to parallel degradation in late endosomes/multivesicular bodies (LE/MVB) through a pathway referred to as endosomal microautophagy (eMI) [8]. This alternative process contributes to the in bulk degradation of cytosolic proteins trapped in vesicles forming at the LE membrane. Interestingly, some proteins bearing a KFERQ domain can also be selectively recognized and degraded by eMI through a LAMP2A-independent mechanism but are in need of the “Endosomal Sorting Complex Required for Transport” (ESCRT) machinery (Figure 1) [9].

Briefly, after being recognized by HSC70 (a common step between CMA and eMI), the substrates targeted by eMI are then transported to the LE/MVB after the direct binding of HSC70 to phosphatidylserine residues on the LE/MVB membrane [10]. Members of the ESCRT machinery, including VPS4 and TSG101, then gather around this area and form a membrane invagination internalizing the substrate into intraluminal vesicles [8]. eMI substrates might then be degraded either within the LE/MVB itself (i.e., Tau [11]) or upon subsequent fusion with a lysosome [8,11]. Alternatively, eMI could also act as a gateway for extracellular protein release due to the ability of LE/MVB to fuse with the plasma membrane and release their luminal content (exosomes) [12]. However, in this latter case, the fate of the cargo is different. Thus, it has been proposed to use the term eMI only for cytosolic proteins loaded in the LE/MVB that undergo degradation in this compartment or during lysosomal fusion and not for those targeted for extracellular release by exocytosis [9]. The characterization of the eMI now raises several questions about the relationship and interplay between CMA and eMI. In particular, the mechanisms underlying the routing of KFERQ-containing substrates to one or the other of these degradation pathways remain to be determined. The respective roles and functional complementarities/overlapping/compensation of these pathways also need to be considered.

Notwithstanding, to date, the co-existence of these two autophagic pathways has only been demonstrated in mammals, and factually, this scenario does not seem to apply to all species. Indeed, the recent discovery that the *LAMP2* gene (encoding LAMP2A) emerged at the root of the vertebrate lineage [13] indicates that invertebrates are de facto unable to perform any mammalian-type of CMA activity [7], or at least a LAMP2A-dependent CMA process such as that recently described in fish [13]. As such, although flies (*Drosophila melanogaster*) appear to be a CMA-incompetent species, they nevertheless perfectly cope with eMI only [14], meaning that eMI could fulfill all the functions shared with CMA in mammals. Interestingly, in fission yeast (*S. pombe*), Liu et al. reported the existence of an ESCRT-dependent eMI-like process that relies on the NBR1 protein (which shares a partial homology with the mammalian macroautophagy receptor NBR1) to deliver two hydrolytic enzymes (LAP2 and APE2) from the cytosol to the vacuole [15]. However, unlike the eMI process described in mammals and *D. melanogaster*, the yeast alternative, termed NBR1-mediated vacuolar targeting (NVT), singularly relies on the ubiquitination of NBR1 and/or its associated proteins and not on HSC70, suggesting that NVT and eMI are indeed two distinct mechanisms. Considering this data, it is tempting to build a scenario in which eMI and CMA would have appeared sequentially during the course of evolution. The oldest eukaryotic lineages would be eMI- and CMA- incompetents, invertebrates (ecdysozoans and lophotrochozoans) would barely perform eMI, and only vertebrates would use both of the pathways, eMI and CMA. To go a step further, the apparent sequence variability of LAMP2A in vertebrates (particularly in its cytosolic C-terminal region that is involved in substrate-chaperone complex recognition) between phylogenetically distant species [16] additionally addresses the possibility of a still ongoing evolution of the mechanisms underlying CMA activity across vertebrates.

In the present review, we summarized the current understanding of the evolutionary history of the main components necessary for CMA and eMI functions and discussed how the observed disparities between species can improve our knowledge of these two functions and their interplays from an evolutionary perspective.

## 2. The Essentials of eMI and CMA

### 2.1. The KFERQ Motif

**Figure 2:**
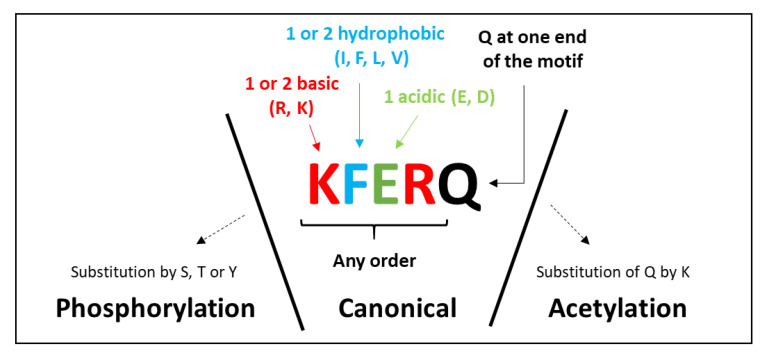
Building rules of canonical, phosphorylation-, and acetylation-generated KFERQ-like motifs (adapted from [21]). The KFERQ-like motif may contain up to two hydrophobic residues (isoleucine (I), phenylalanine (F), leucine (L), or valine (V)), up to two positive residues (arginine (R) or lysine (K)), and a single negatively-charged residue (glutamate € or aspartate (D)) flanked at either the N- or C-terminus of the pentapeptide by a single glutamine (Q) residue. KFERQ-like motifs can also be generated via post-translational modifications, such as phosphorylation or acetylation of the amino acid residues along protein regions that do not originally show the mentioned properties.

A common and essential step between eMI and CMA is the recognition by HSC70 of the KFERQ-like motif-containing proteins. The existence of either of these processes (eMI or CMA) in a given species, therefore, requires the presence of proteins bearing this motif or, at least, a sequence related to this motif, but how was this motif defined? Pioneering studies performed by Fred J Dice using bovine pancreatic ribonuclease A (RNase A) initially identified an 11-amino acid region within the protein, and later on, this motif was narrowed down to the pentapeptide KFERQ, which is necessary and sufficient to target proteins for lysosomal degradation [16]. However, the exact sequence, -KFERQ-, is only contained in RNase A. Further studies conducted by the same team also demonstrated that the physical properties of the residues constituting the motif, rather than the specific amino acids per se, determine the ability of the chaperone HSC70 to bind this region [17]. Accordingly, these authors defined that a canonical KFERQ-like motif is always flanked by a glutamine (Q) on either side and must contain (i) one or two of the positive residues K and R, (ii) one or two of the hydrophobic residues F, L, I, or V, and (iii) one of the negatively-charged residues E or D (Figure 2). Recently, other studies have demonstrated that KFERQ-like motifs can be generated via post-translational modifications. Thus, phosphorylation or acetylation of the amino acid residues along protein regions that do not originally display the mentioned properties dramatically increases the repertory of putative HSC70 substrates (Figure 2) [18,19,20].

According to the above-described definition of the KFERQ-like motif, approximately 46% of proteins in human proteome contain at least one canonical motif, 20% contain no canonical motif but a phosphorylation-generated one, and 9% contain only acetylation-generated motifs [22]. Interestingly, the overall percentages of proteins bearing each of the KFERQ-like-types motifs are similar between the proteome of *Mus musculus* (which is both CMA- and eMI-competent), *Drosophila melanogaster* (which is eMI-competent but CMA-incompetent), and *Saccharomyces cerevisiae* (unable to perform CMA and whose ability to perform a “mammalian-type” eMI has not been demonstrated) [22]. These results suggest that the emergence of the KFERQ-like motif most likely preceded the establishment of the CMA function (and possibly that of eMI as well) and that the proportion of these proteins in the proteome of a given species is thus not predictive of its ability to perform any of these selective autophagy pathways. Nonetheless, a recent comparison of more than 500 orthologs of human proteins harboring a single canonical KFERQ motif in 50 different species predicted to be either CMA-competent or -incompetent, revealed that about 45% of the investigated proteins displayed a more preserved motif in “CMA-proficient” species than in “CMA-deficient” ones [22]. This suggests a substantial degree of evolution of this motif in different species. Whether the evolution of this region is dependent on their ability to perform either eMI, CMA, or both remains to be further investigated.

### 2.2. HSC70

The chaperone HSC70, a member of the heat shock protein 70 family (HSP70), is mostly constitutively expressed. It is involved in diverse cellular processes, including protein folding and protein degradation. We do not intend here to give a detailed description of all the various functions fulfilled by this protein. It has already been well described in excellent reviews that we encourage to consult [23,24,25]. Instead, we will rather discuss the evolutionary history of this chaperone and draw up a scenario that highlights how the two autophagic functions (eMI and CMA) might have specifically evolved, although using/sharing an initial common component.

Proteins of the HSP70 family appeared very early during evolution and are found in a wide range of living organisms, from archaea to mammals [26,27,28]. Interestingly, significant differences in the number of genes belonging to this family are clearly visible between species. Thus, a recent screen for the presence of HSP70 in 5551 representative prokaryotic genomes revealed that the number of HSP70 genes in individual prokaryotic genomes ranges from 0–23 (99.1% of the genomes contain at least one HSP70 gene) [29]. Similarly, it is well known that most eukaryotic cells also display several genes coding for HSP70/HSC70 proteins. For example, the yeast *Saccharomyces cerevisiae* has more than 10 genes coding for proteins of this family [30], which have all been shown to present both overlapping and divergent functions [31]. Furthermore, at least 12 genes encoding 14 proteins of the HSP70 family are found in the human genome [23]. Although all of them display an overall high sequence identity (more than half share 80% of their amino acid sequence), these proteins are nevertheless likely involved in distinct functions due to, notably, specific spatio-temporal distributions [23].

**Figure 3:**
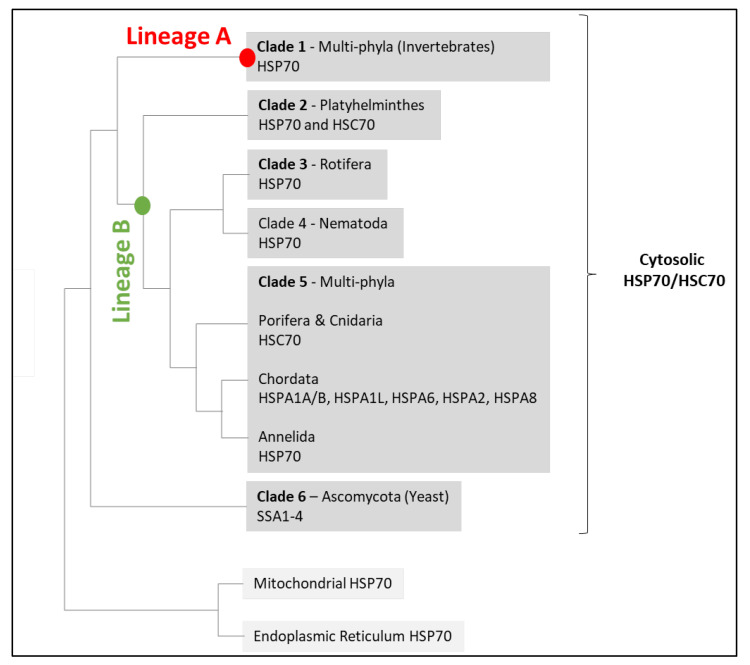
Proposed tree topology of HSP70/HSC70 family members in metazoans (adapted from Yu et al. [32]). Red and green circles show the nodes that give birth to Lineage A of invertebrate HSP70s (clade 1) and Lineage B of both vertebrate and invertebrate HSP70s and all HSC70 genes, respectively. This second lineage has further diversified within diverse phyla: *Platyhelminthes* (clade 2), *Rotifera* (clade 3), *Nematoda* (clade 4), and *Chordata* (clade 5). Clades 1 to 6 include only cytosolic members of the HSP70 family.

Most of the HSP70 family members are cytosolic proteins. They have further been classified into either inducible HSP70s or constitutively expressed HSC70s, but until recently, their evolutionary relationship remained elusive, mainly due to the lack of cross-phylum comparisons. However, a recent in-depth phylogenetic study reviewing 125 complete HSP70/HSC70s genes from a wide range of species across different phyla has clarified the complex evolution of this gene family [32]. According to this study, HSP70 family members associate with mitochondria and the endoplasmic reticulum form two monophyletic groups (Figure 3). Members of the cytosolic HSP70 family form a third monophyletic group, which also includes yeast cytosolic HSP70s (with both inducible and constitutive ssa1–4 genes). Within metazoan cytosolic HSP70/HSC70s, two large lineages are distinct. These two lineages most certainly formed before the separation of vertebrates and invertebrates. One lineage includes a relatively limited number of genes from many invertebrate phyla, none of which have been shown to be constitutively expressed. The second lineage contains both inducible and constitutive genes from various phyla. This second lineage has further diversified within some phyla (including at least *Platyhelminthes*, *Rotifera*, *Nematoda*, and *Chordata*). In this regard, some genes from the second lineage have certainly either gained or lost their stress-inducible response capacity (through convergent evolution), which may explain the sporadic distribution of “HSP70” and “HSC70” in previous phylogenetic analyses. However, due to the presence of inducible and constitutive HSP70 gene members in many clades (including yeast), it is still difficult to predict the ancestral state.

Interestingly, an inter-clade comparison of the synonymous and nonsynonymous substitution rates revealed that all HSP70/HSC70 family members are under strong purifying selection [32]. The presence of such a purifying selection was already reported for mammalian [33], nematode [34], molluscan [35], and protist [36] HSP70/HSC70 genes. That positive selection would most likely operate to preserve functions performed by HSP70s, including heat shock response, folding of newly synthesized proteins, protein transport, and autophagy.

Overall, this set of data supports an ancient origin of the chaperone HSC70, and its relatively high sequence conservation between phyla suggests preserved functions during the evolution. This being said, one might be tempted to speculate that the presence of both KFERQ-containing proteins and orthologs of HSC70 in yeast (i.e., ssa1p) signs a hallmark of an ancient origin of eMI and/or CMA. However, these two autophagic pathways also rely on other factors, such as members of the ESCRT machinery (in the case of eMI) and LAMP2A (in the case of CMA). Hence, a critical question is whether these factors also appeared very early during evolution or whether eMI/CMA functions formed later on from pre-existing and evolutionary conserved components.

## 3. Endosomal Microautophagy

After its initial description and characterization in mammals in the late 1980s [37,38], most progress in the microautophagy area has been achieved by research made in yeast, ranging from the microautophagy of peroxisomes [39,40], mitochondria [41], portions of nuclei [42], lipid droplets [43], endoplasmic reticulum [44], certain cytosolic enzymes [15], and vacuole membrane proteins [45]. Altogether, these findings provided evidence that the microautophagy function emerged very early during the evolution and that it can virtually target any cellular structure. However, the diversity of the mechanisms involved in handling/processing these different cellular constituents has led many authors to wonder about the relevance of gathering these distinct types of autophagy under the same “microautophagy” process(es) (or at least terminology) [46].

The present review focused on a specific “subtype” of microautophagy that was first characterized in murine dendritic cells [8]. This process, termed eMI, contributes to in bulk degradation of proteins present in the cytosol and that are embedded in vesicles formed from the LE membrane. However, some cytosolic proteins can also be selectively degraded by eMI after HSC70 recognizes their KFERQ motifs and binds to endosomes via phosphatidylserine [8]. Members of the ESCRT machinery (including at least VPS4 and TSG101) then induce membrane invagination and subsequent internalization of the substrates into intraluminal vesicles [8]. Cargo degradation then occurs either in the LE/MVB compartment or after the fusion of LE/MVB with lysosomes.

More recently, the existence of a similar eMI process was described in the fat body of flies [14]. Akin to mammals, components of the ESCRT machinery have been proven to be necessary for eMI in *Drosophila*. However, in contrast to mammals, eMI in flies involves additional autophagy-related genes (such as *ATG1* and *ATG13*) and is induced after fasting [14]. Of note, genotoxic and oxidative stresses have also been shown to up-regulate eMI in *Drosophila* [47]; although in these cases, the dependence on HSC70 is only partial, pointing out the importance of carefully discriminating between HSC70-dependent and HSC70-independent types of eMI.

Although an HSC70-dependent type of eMI has attracted much attention after it was just discovered, very few data are actually available regarding its regulation and functional implications compared to other forms of autophagy. To our knowledge, this particular mechanism has only been documented in mice [8] and *Drosophila* [14,48]. It is still unclear whether this singular autophagic pathway exists in other living organisms. Interestingly, extensive comparative genomic and phylogenetic studies have demonstrated that ESCRT genes are conserved throughout the eukaryotic lineage [49], as well as in Archaea with respect to some genes [50,51]. That broad distribution in eukaryotes (and some Archaea) supports the early evolutionary origin of the ESCRT machinery and by inference, possibly, of an eMI-like pathway similar to the one described in mice and *Drosophila*. In this context, Liu et al. reported the existence of an ESCRT-dependent eMI-like process relying on the NBR1 protein in fission yeast (*S. pombe*) [15]. However, while this pathway has been proven to be dependent on the ubiquitination of NBR1 and/or its associated proteins, it does not involve HSC70.

## 4. Chaperone-Mediated Autophagy

CMA activity is tightly correlated with (i) the amount of LAMP2A at the lysosomal membrane [52] and (ii) the assembly/disassembly efficiency of LAMP2A complexes in this compartment [5]. As such, analyzing either the presence/absence of the gene coding for that protein, or the variability observed within the different functional protein domains between phylogenetically distant species, would certainly help decipher the evolutionary history of this function.

### 4.1. Origin and Evolution of LAMP2

**Figure 4:**
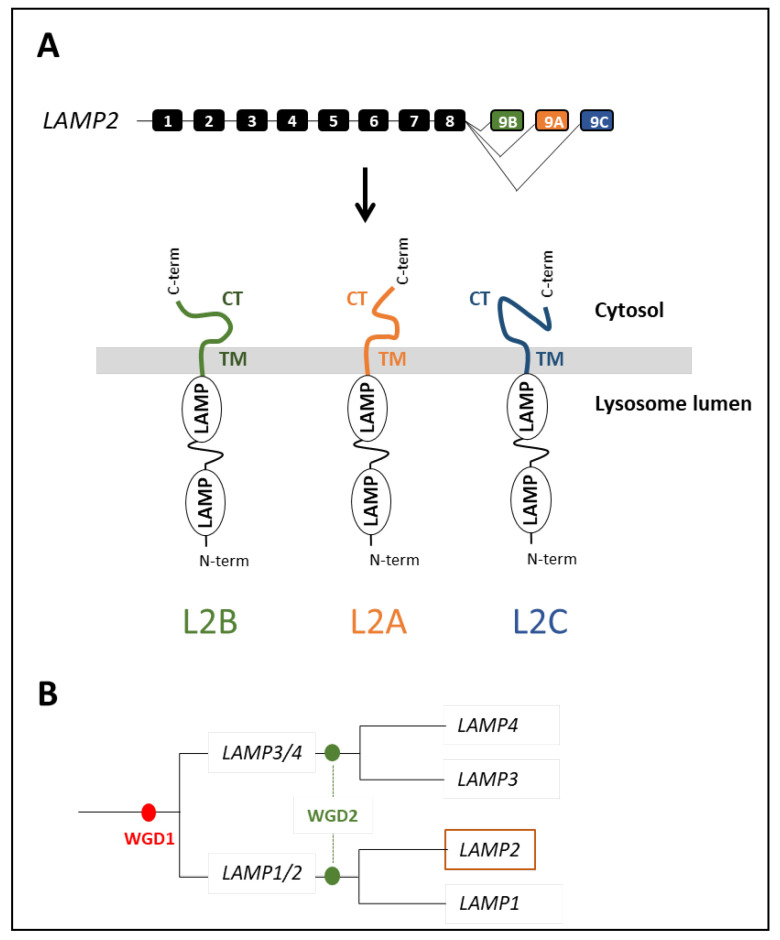
(**A**) Gene organization of the gene *LAMP2* and protein structure of three splice variants displaying a common luminal domain (with two LAMP domains) but different transmembrane (TM) domain and cytosolic tail (CT). The boxed numbers denote exons. (**B**) Schematic scenario of the evolutionary history of lamp family genes.

LAMP2A originates from the alternative splicing of the *LAMP2* gene, which also generates two other different splice variants, LAMP2B and LAMP2C. While these three splice variants all share a common luminal domain, they also display different and specific cytosolic and transmembrane™ regions (Figure 4A) [53,54]. In contrast to LAMP2A, neither LAMP2B nor LAMP2C has been shown to be involved in CMA. Instead, LAMP2B is involved in macroautophagy [55,56] and the LAMP2C in the uptake and degradation of DNA and RNA molecules by lysosomes [57,58].

Recently, Lescat et al. provided a comprehensive picture of the evolutionary history of the *LAMP2* gene in vertebrates. They demonstrated that *LAMP2* appeared after the second round of whole-genome duplication (WGD) at the root of the vertebrate lineage, ~500 Ma [13]. More precisely, phylogenetic analyses and synteny conservation data strongly suggest that a single copy of *LAMP* was already present in the common vertebrate ancestor, and that the two successive WGDs that occurred at the root of the vertebrate lineages [59] engendered *LAMP1/2* and *LAMP3/4* (from WGD1), and then *LAMP1*, *LAMP2*, *LAMP3*, and *LAMP4* (from WGD2) that are common to all vertebrates (Figure 4B). Hence, data support the idea that invertebrates would not factually be able to perform CMA, or at least a LAMP2A-dependent CMA process similar to the one described in mammals [7] and fish [13].

**Figure 5:**
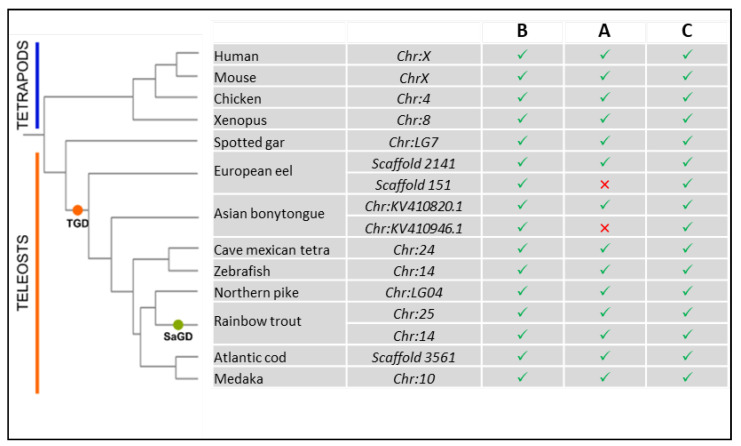
Presence of the three alternative exons (**B**, **A**, and **C**) in the *LAMP2* gene in four tetrapod species—human (*H. sapiens*), mouse (*M. musculus*), chicken (*G. gallus*), and frog (*X. tropicalis*)—and nine fish species—spotted gar (*L. oculatus*), European eel (*A. anguilla*), Asian bonytongue (*S. formosus*), cave Mexican tetra (*A. mexicanus*), zebrafish (*D. rerio*), Northern pike (*E. lucius*), rainbow trout (*O. mykiss*), Atlantic cod (*G. morhua*), and medaka (*O. latipes*). The phylogenetic tree is a representative tree of life. The presence or absence of each alternative exon (**B**, **A**, and **C**) is represented by a green check mark or a red cross mark, respectively. For each species, the location of the gene is specified in the table. TGD, teleost-specific whole-genome duplication; SaGD, salmonid-specific whole-genome duplication.

It is also worth noting that, despite the significant genomic rearrangements that some vertebrate lineages have experienced [60], the number of *LAMP2* genes is constant across vertebrate genomes. Indeed, the third WGD, which occurred 320–350 million years ago in the teleost fish ancestor (namely, the teleost-specific round of WGD or TGD), theoretically implies that two orthologs (co-orthologs) of each human *LAMP* gene would be expected in teleost species, unless lost. Accordingly, both the European eel (*Anguilla anguilla*) and the Asian bonytongue (*Scleropages formosus*), whose lineages diverged shortly after the TGD, display two *LAMP2* genes, with one paralog bearing the three alternative exons and the other only the exons B and C [13] (Figure 5).

The absence of exon A in the second *LAMP2* paralog of these two species, belonging to different super-orders, could be due to either independent losses or the specific loss of this exon in the common ancestor of teleost shortly after the TGD. However, this second *LAMP2* gene, which likely results from the TGD, appears to have been lost in all other teleost species investigated [13]. Accordingly, in cave Mexican tetra (*Astyanax mexicanus*), zebrafish (*Danio rerio*), Northern pike (*Esox lucius*), Atlantic cod (*Gadus morhua*), and medaka *(Oryzias latipes)*, a unique *LAMP2* is found with all three alternative exons (Figure 5). Interestingly, genome analyses of various salmonid species have revealed the presence of two *LAMP2* genes bearing the three alternative exons A, B, and C, which likely originated from the fourth round of WGD that occurred in the common ancestor of salmonids (SaGD) about 100 million years ago. However, a re-analysis of the PhyloFish RNA-seq database—providing gene expression data from 23 different ray-finned fish species [61]—confirmed the expression of a single LAMP2A transcript in all considered salmonids [16]. This suggests that the loss of expression of the second LAMP2A transcript is of a common origin, probably in the ancestor of salmoniforms, shortly after the SaGD. Together, these data suggest that evolution tends to promote the presence of a single LAMP2A protein. Indeed, duplication of the *LAMP2* genes following WGD appears to be systematically followed by the loss of one duplicate, the loss of the A exon, or the lack of expression of one of the two copies (in the case of the recent SaGD). This last point certainly deserves to be clarified in the future.

### 4.2. Structure Evolution of LAMP2A across Vertebrates

In mammals, the structure of the lysosomal membrane proteins belonging to the LAMP family is now well documented [62,63,64,65,66]. These proteins consist of a large luminal region at the N-terminus, a TM domain of about 20 amino acids, and a short Cytosolic (C-terminal) Tail (CT) stretching from 10–12 amino acids (Figure 4A). In addition, these proteins possess a number of conserved motifs required for protein lysosomal targeting and function (see [5,52,67,68] for details). Although the general architecture of this protein is relatively well conserved among vertebrates, some differences are noticed within the different functional domains of LAMP2A between phylogenetically distant species. Taking advantage of that “evolution in motion” will certainly be of great value for identifying evolutionarily conserved, or species-dependent, key residues necessary for further deciphering the structure–function relationship of this protein.

#### 4.2.1. The GYXXϕ Motif

**Figure 6:**
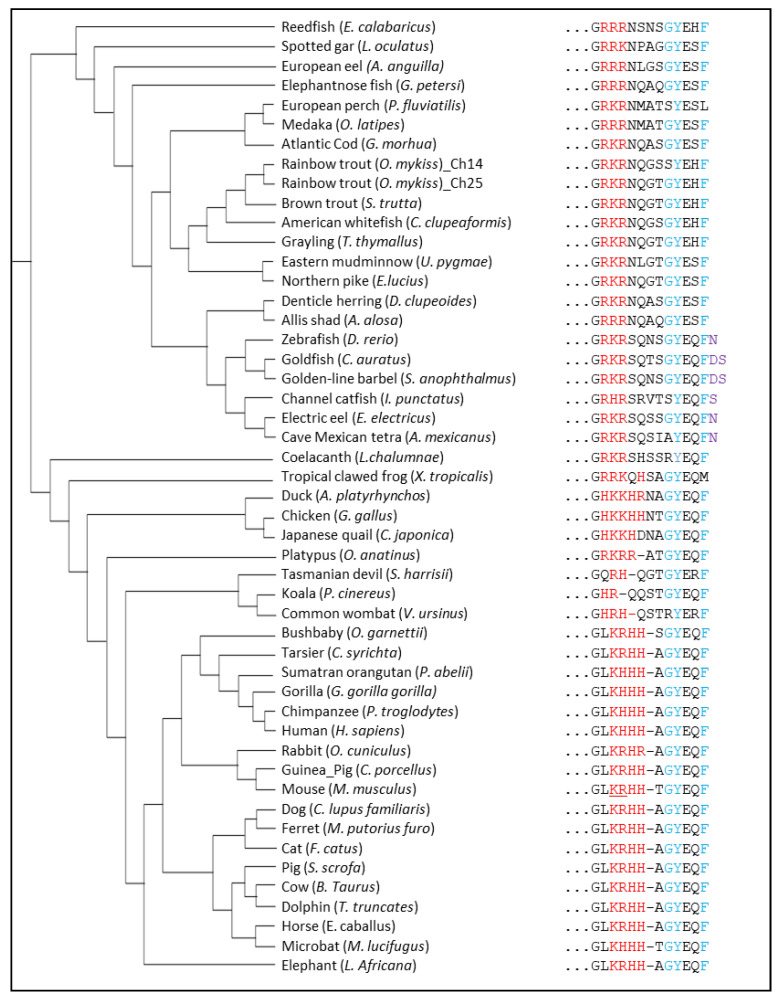
Protein sequence alignment of the short CT tail of LAMP2A from different vertebrates. Positively-charged amino acids required for the binding of substrate proteins are colored in red. The GY dipeptide as well as the hydrophobic F required for the targeting of LAMP2A to lysosomes are in blue. The additional residues at the C-terminus of LAMP2A from fish belonging to ostariophysans are in purple. The K406 and R407 of mouse LAMP2A are underlined.

According to the literature, the C-terminal ends of LAMP proteins carry a recognition signal for lysosomal targeting, which is characterized by the canonical GYXXϕ motif (where ϕ is a hydrophobic amino acid) [52]. In mammalian LAMP2A, ϕ is the hydrophobic phenylalanine (F) residue, whose deletion has been shown to impair the proper addressing of LAMP2A to the lysosomal membrane [52]. Further analysis of LAMP2A sequences additionally revealed that this hydrophobic F residue is actually highly conserved in vertebrates (Figure 6). Excepting the absence of that “F” in *Xenopus tropicalis* and *Perca fluviatilis*, the only variations identified are the presence of one or two extra amino acids at the C-terminus in some fish species belonging to ostariophysans (a superorder comprising about 8000 species of bony fish, including catfishes, characins, electric knifefishes, and the zebrafish). In addition, recent findings showing that extra amino acids (i.e., a triple FLAG tag) at the C-terminus of LAMP2A do not impair neither its lysosomal addressing nor its ability to properly target well-known CMA substrates (such as GAPDH) fused to the HaloTag protein (GAPDH-HT) [62] indicate that these extra C-terminus residues are undeniably not crucial for a proper CMA function.

It should also be noted that, while in the majority of the vertebrate species analyzed the GY dipeptide is conserved when focusing on the cytosolic tail sequence of LAMP2A, it is nevertheless not “retained” in some (e.g., in the common wombat (*Vombatus ursinus*), the blind cave tetra (*Astyanax mexicanus*), the coelacanth (*Latimeria chalumnae*), the channel catfish (*Ictalurus punctatus*), the European perch (*Perca fluviatilis*), and one of the two LAMP2A found in the rainbow trout) (Figure 6). These data might suggest that LAMP2A in these species is (possibly) not correctly targeted to the lysosomal membrane. However, previous studies carried out on the LAMP1 protein (human and mouse), which also presents the GY dipeptide, clearly demonstrated that only tyrosine (Y) is required for the localization of the protein at the lysosomal membrane [67,68]. In contrast, while mutating the glycine does not impair the correct addressing of LAMP1 to the lysosomal membrane [67], on the other hand, it does affect its routing to the lysosomes [69,70].

Together, these data suggest that the LAMP2A proteins, as far as the large majority of the vertebrates is concerned, do require the presence of the minimal YXXF motif in their cytosolic tails to be correctly addressed to the lysosomal membrane.

#### 4.2.2. Positively-Charged Amino Acids

Although the presence of the YXXF motif identified within the cytosolic tail of the LAMP2A from most vertebrate species suggests that these proteins can, in theory, be properly delivered to the lysosomal membrane, the activity of this protein also relies on several other motifs nested within the C-terminal region. In mammals, it is commonly accepted that the presence of 3–4 positively charged residues within the cytosolic tail of LAMP2A is necessary for the proper recognition and binding of the substrate/chaperone complex [52]. In that direction, isolated lysosomes containing a mutated version of LAMP2A for the four positive residues displayed a lower binding ability against GAPDH when compared to native LAMP2A residues [52].

Very recently, using a site-specific photo-reactive crosslinking experiment, Ikami et al. evidenced the direct interaction of the cytoplasmic tail of mouse LAMP2A together with HSC70 [66]. More precisely, a UV-dependent crosslinking was observed at two positively charged amino acids (K406 and R407 of mouse LAMP2A), which are located immediately after the TM region (underlined residues for the mouse sequence in Figure 6). This finding supports the conclusions drawn after monitoring by nuclear magnetic resonance (NMR)-based chemical shift perturbation (CSP) assay of the interactions between human TM-LAMP2A (369–410, consisting of the transmembrane domain and cytoplasmic tail) and the substrate-binding domain of HSC70 [64]. In the presence of the substrate-binding domain of HSC70, residues K401 and H402 (corresponding to K406 and R407 in mouse LAMP2A, respectively) showed significant CSPs, while those of the TM residues were essentially unchanged [64]. Together, these findings support an intrinsic affinity of HSC70 for the cytoplasmic tail of LAMP2A and an important role of the two positively charged residues in this affinity. Of note, a similar pattern of CSPs was also observed when a CMA substrate, RNase A, was added to TM-LAMP-2A [64], further emphasizing the role of these residues in substrate affinity and specificity.

Interestingly, sequence analysis of vertebrates LAMP2A shows the presence (in the majority of the species considered) of at least three positively charged amino acids (including the two amino acids corresponding to K406 and R407 in mouse LAMP2A) (Figure 6). These results suggest that, in addition to bearing the lysosome membrane addressing motifs discussed above (see Section 4.2.1), the analyzed LAMP2A from most of the vertebrates also carry the motifs necessary for substrate recognition and binding.

#### 4.2.3. Glycine Residues in the Transmembrane Region

The GAALAG motif, located at the TM region of LAMP2A (382–387 in *H. sapiens*), is known to be involved in dimerization. More precisely, it is important for the oligomerization of LAMP2A and, consequently, for the translocation of target proteins inside lysosomes [71]. Site-directed mutagenesis of the two glycine (G) residues to alanine (A) has been shown to impair the oligomerization of LAMP2A and, by implication, substrates translocation [5,64,71]. Recently, an in-depth analysis of the coding sequences of LAMP2A from 45 mammalian species belonging to the nine major placental orders (*Chiroptera*, *Carnivora*, *Perissodactyla*, *Artiodactyla*, *Soricomorfa*, *Erinaceomorpha*, *Primates*, *Lagomorpha*, and *Rodentia*) and three marsupials reported that these two glycines are indeed conserved across mammals [72]. While few exceptions should be noticed in the star-nosed mole (*C. cristata*), for which the second glycine is substituted by an isoleucine, in two megabats, *P. alecto* and *P. vampyrus*, as well as in the *Muridae* family of rodents, *M. musculus*, *M. pahari*, *R. norvegicus*, and *M. caroli*, species for which the motif is converted to GTALAG and GAALGG, respectively, it is not clear whether these variations do significantly affect the dimerization and function of LAMP2A. Nevertheless, the overall high preservation of the GAALAG motif within the vertebrate clade would support purifying selection during evolution.

Two glycines are also found in the TM region of LAMP2A in three fish species analyzed (spotted garfish, European eel, and arowana). However, one of these two glycines are absent in all other fish considered in our analyses, supporting the existence of functional variation of LAMP2As between species. Nevertheless, effects related to a single glycine mutation have so far not been reported.

#### 4.2.4. The LAMP Domains

**Figure 7:**
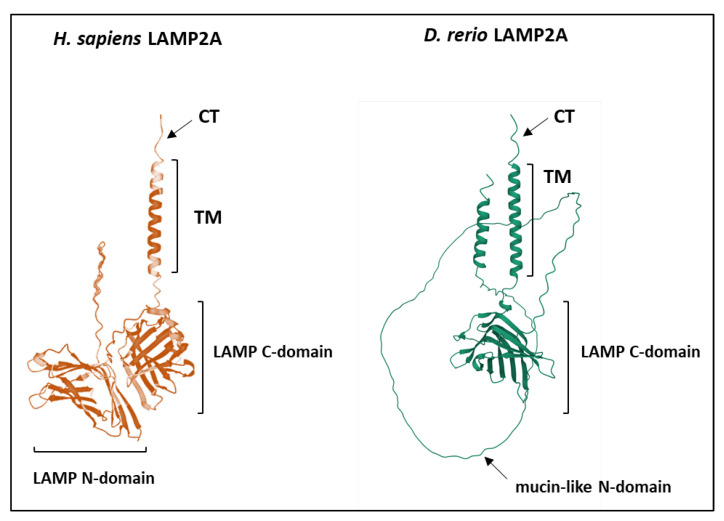
Structure prediction of *Homo sapiens* and *Danio rerio* LAMP2As showing the strong structural divergence of the two proteins at the membrane-distal LAMP domain (Source: AlphaFold Protein Structure Database [73,74]). Instead of a beta-sheet structure classically observed in most vertebrates, the zebrafish N-domain exhibits a mucin-like structure. CT, cytoplasmic tail; TM, transmembrane domain; LAMP C-domain, membrane-proximal LAMP domain; LAMP N-domain, membrane-distal LAMP domain.

In mammals, all LAMP2 proteins (B, A, and C) display two conserved domains—the LAMP domains—being both located in the intra luminal compartment of the lysosomes (Figure 4A). Data reporting on the possible role(s) of these two domains (N- and C- domains) are sparse, but a recent study suggests that the N-domain may act as a negative regulator of the (self)multimerization step between LAMP2 proteins [65]. Interestingly, sequence analysis of the different LAMP2As retrieved from the above-mentioned [72] placental mammals clearly indicates that the N-domain is less conserved compared to the second luminal C-domain. The same holds when compared to the TM and CT regions. These results suggest that a relaxation of the purifying selection may have occurred for this distal luminal domain, allowing it to harbor functional diversity during mammalian evolution. In this regard, it is noteworthy that, in fish species belonging to the Ostariophyses (including zebrafish), this luminal N-domain displays major structural variations (Figure 7), further supporting the possibility that it may play an important role accounting for the functional variation of LAMP2A between vertebrates. However, further functional analyses will be required to support this hypothesis.

Overall, these data show that the sequences of LAMP2As from different vertebrates contain the motifs necessary for (i) addressing the lysosomal membrane, (ii) recognizing the substrates to be degraded, and (iii) acquiring the conformation necessary for translocation across the lysosomal membrane. It thus suggests that most vertebrates may have a CMA (or CMA-like) activity similar to that described in humans and mice (and recently in fish). However, depending on the species considered, these motifs nevertheless harbor numerous variations compared to the “canonical” human or mice LAMP2As. Which residues are dispensable, required, or absolutely crucial for the proper functioning of LAMP2A remain to be functionally tested.

## 5. Concluding Remarks and Pending Questions

In mammals, the coexistence of CMA and eMI, both of which rely on the recognition of a KFERQ-like motif by the chaperone HSC70, raises many questions dealing with the respective roles of these two pathways and their interplay [9]. However, this dichotomic picture does not seem to apply to all species, and the presence of only one of both functions, or even none of them, has already been reported in some organisms [22]. Such a diversity of situations offers the opportunity to better appreciate the respective role(s) of each of these functions independently and from a comparative/evolutionary point of view. However, it also raises fundamental questions about the evolutionary history of these two functions: when did they appear? How did they evolve? How did they conjointly evolve? Can they functionally substitute each other?

Overall, the data we aggregated in the present review provide unequivocal evidence that the *LAMP2* gene appeared at the onset of vertebrate radiation [13], de facto indicating that CMA (as currently described) is specific to this lineage. However, the existing sequence variability within the different functional domains of LAMP2A between phylogenetically distant vertebrates may suggest that CMA function has evolved differently between species, ultimately resulting in a variety of different cellular/physiological functions. In the future, the study of the structure/function relationship of LAMP2A in diverse vertebrates will certainly help decipher the functional importance of the several different domains of this protein and, more generally, will provide new insights into the species-specificities of this function in non-mammalian species.

The origin and the evolutionary trajectory of eMI are not that straightforward. Indeed, although most of the core proteins/components of eMI (including the chaperone HSC70, KFERQ-motif containing proteins, as well as major components of the ESCRT machinery) are already present at the onset of eukaryotic evolution, functional validations of this function (as currently defined) have only been provided for mice and *Drosophila* [8,14]. On the other hand, the existence of an ESCRT-dependent eMI-like process has been reported in fission yeast (*S. pombe*), arguing for a much more ancient origin of this process [15]. However, and unlike the eMI process described in mammals and *Drosophila*, the yeast pathway does not rely on HSC70, suggesting that these should be considered as two distinct mechanisms. Hence, the question of the origin and evolution of the eMI still remains open and will certainly provide new exciting avenues for autophagy research in the next coming years.

## Data Availability

Not applicable.

## References

[1] Chiang H.L., Dice J.F. (1988). Peptide Sequences That Target Proteins for Enhanced Degradation during Serum Withdrawal. J. Biol. Chem..

[2] Chiang H.L., Terlecky S.R., Plant C.P., Dice J.F. (1989). A Role for a 70-Kilodalton Heat Shock Protein in Lysosomal Degradation of Intracellular Proteins. Science.

[3] Agarraberes F.A., Dice J.F. (2001). A Molecular Chaperone Complex at the Lysosomal Membrane Is Required for Protein Translocation. J. Cell Sci..

[4] Cuervo A.M., Dice J.F. (1996). A Receptor for the Selective Uptake and Degradation of Proteins by Lysosomes. Science.

[5] Bandyopadhyay U., Kaushik S., Varticovski L., Cuervo A.M. (2008). The Chaperone-Mediated Autophagy Receptor Organizes in Dynamic Protein Complexes at the Lysosomal Membrane. Mol. Cell. Biol..

[6] Bandyopadhyay U., Sridhar S., Kaushik S., Kiffin R., Cuervo A.M. (2010). Identification of Regulators of Chaperone-Mediated Autophagy. Mol. Cell.

[7] Kaushik S., Cuervo A.M. (2018). The Coming of Age of Chaperone-Mediated Autophagy. Nat. Rev. Mol. Cell Biol..

[8] Sahu R., Kaushik S., Clement C.C., Cannizzo E.S., Scharf B., Follenzi A., Potolicchio I., Nieves E., Cuervo A.M., Santambrogio L. (2011). Microautophagy of Cytosolic Proteins by Late Endosomes. Dev. Cell.

[9] Tekirdag K., Cuervo A.M. (2018). Chaperone-Mediated Autophagy and Endosomal Microautophagy: Joint by a Chaperone. J. Biol. Chem..

[10] Morozova K., Clement C.C., Kaushik S., Stiller B., Arias E., Ahmad A., Rauch J.N., Chatterjee V., Melis C., Scharf B. (2016). Structural and Biological Interaction of Hsc-70 Protein with Phosphatidylserine in Endosomal Microautophagy. J. Biol. Chem..

[11] Caballero B., Wang Y., Diaz A., Tasset I., Juste Y.R., Stiller B., Mandelkow E.-M., Mandelkow E., Cuervo A.M. (2018). Interplay of Pathogenic Forms of Human Tau with Different Autophagic Pathways. Aging Cell.

[12] Caballero B., Bourdenx M., Luengo E., Diaz A., Sohn P.D., Chen X., Wang C., Juste Y.R., Wegmann S., Patel B. (2021). Acetylated Tau Inhibits Chaperone-Mediated Autophagy and Promotes Tau Pathology Propagation in Mice. Nat. Commun..

[13] Lescat L., Véron V., Mourot B., Péron S., Chenais N., Dias K., Riera-Heredia N., Beaumatin F., Pinel K., Priault M. (2020). Chaperone-Mediated Autophagy in the Light of Evolution: Insight from Fish. Mol. Biol. Evol..

[14] Mukherjee A., Patel B., Koga H., Cuervo A.M., Jenny A. (2016). Selective Endosomal Microautophagy Is Starvation-Inducible in Drosophila. Autophagy.

[15] Liu X.-M., Sun L.-L., Hu W., Ding Y.-H., Dong M.-Q., Du L.-L. (2015). ESCRTs Cooperate with a Selective Autophagy Receptor to Mediate Vacuolar Targeting of Soluble Cargos. Mol. Cell.

[16] Lescat L., Herpin A., Mourot B., Véron V., Guiguen Y., Bobe J., Seiliez I. (2018). CMA Restricted to Mammals and Birds: Myth or Reality?. Autophagy.

[17] Dice J.F. (1990). Peptide Sequences That Target Cytosolic Proteins for Lysosomal Proteolysis. Trends Biochem. Sci..

[18] Lv L., Li D., Zhao D., Lin R., Chu Y., Zhang H., Zha Z., Liu Y., Li Z., Xu Y. (2011). Acetylation Targets the M2 Isoform of Pyruvate Kinase for Degradation through Chaperone-Mediated Autophagy and Promotes Tumor Growth. Mol. Cell.

[19] Kaushik S., Cuervo A.M. (2015). Degradation of Lipid Droplet-Associated Proteins by Chaperone-Mediated Autophagy Facilitates Lipolysis. Nat. Cell Biol..

[20] Bonhoure A., Vallentin A., Martin M., Senff-Ribeiro A., Amson R., Telerman A., Vidal M. (2017). Acetylation of Translationally Controlled Tumor Protein Promotes Its Degradation through Chaperone-Mediated Autophagy. Eur. J. Cell Biol..

[21] Jackson M.P., Hewitt E.W. (2016). Cellular Proteostasis: Degradation of Misfolded Proteins by Lysosomes. Essays Biochem..

[22] Kirchner P., Bourdenx M., Madrigal-Matute J., Tiano S., Diaz A., Bartholdy B.A., Will B., Cuervo A.M. (2019). Proteome-Wide Analysis of Chaperone-Mediated Autophagy Targeting Motifs. PLoS Biol..

[23] Stricher F., Macri C., Ruff M., Muller S. (2013). HSPA8/HSC70 Chaperone Protein: Structure, Function, and Chemical Targeting. Autophagy.

[24] Liao Y., Tang L. (2014). The Critical Roles of HSC70 in Physiological and Pathological Processes. Curr. Pharm. Des..

[25] Bonam S.R., Ruff M., Muller S. (2019). HSPA8/HSC70 in Immune Disorders: A Molecular Rheostat That Adjusts Chaperone-Mediated Autophagy Substrates. Cells.

[26] Gupta R.S., Singh B. (1994). Phylogenetic Analysis of 70 KD Heat Shock Protein Sequences Suggests a Chimeric Origin for the Eukaryotic Cell Nucleus. Curr. Biol..

[27] Hunt C., Morimoto R.I. (1985). Conserved Features of Eukaryotic Hsp70 Genes Revealed by Comparison with the Nucleotide Sequence of Human Hsp70. Proc. Natl. Acad. Sci. USA.

[28] Lindquist S., Craig E.A. (1988). The Heat-Shock Proteins. Annu. Rev. Genet..

[29] Pan Z., Zhang Z., Zhuo L., Wan T., Li Y. (2021). Bioinformatic and Functional Characterization of Hsp70s in Myxococcus Xanthus. mSphere.

[30] Werner-Washburne M., Craig E.A. (1989). Expression of Members of the Saccharomyces Cerevisiae Hsp70 Multigene Family. Genome.

[31] Boorstein W.R., Ziegelhoffer T., Craig E.A. (1994). Molecular Evolution of the HSP70 Multigene Family. J. Mol. Evol..

[32] Yu E., Yoshinaga T., Jalufka F.L., Ehsan H., Welch D.B.M., Kaneko G. (2021). The Complex Evolution of the Metazoan HSP70 Gene Family. Sci. Rep..

[33] Hess K., Oliverio R., Nguyen P., Le D., Ellis J., Kdeiss B., Ord S., Chalkia D., Nikolaidis N. (2018). Concurrent Action of Purifying Selection and Gene Conversion Results in Extreme Conservation of the Major Stress-Inducible Hsp70 Genes in Mammals. Sci. Rep..

[34] Nikolaidis N., Nei M. (2004). Concerted and Nonconcerted Evolution of the Hsp70 Gene Superfamily in Two Sibling Species of Nematodes. Mol. Biol. Evol..

[35] Kourtidis A., Drosopoulou E., Nikolaidis N., Hatzi V.I., Chintiroglou C.C., Scouras Z.G. (2006). Identification of Several Cytoplasmic HSP70 Genes from the Mediterranean Mussel (Mytilus Galloprovincialis) and Their Long-Term Evolution in Mollusca and Metazoa. J. Mol. Evol..

[36] Krenek S., Schlegel M., Berendonk T.U. (2013). Convergent Evolution of Heat-Inducibility during Subfunctionalization of the Hsp70 Gene Family. BMC Evol. Biol..

[37] Marzella L., Ahlberg J., Glaumann H. (1981). Autophagy, Heterophagy, Microautophagy and Crinophagy as the Means for Intracellular Degradation. Virchows Arch. B Cell Pathol. Incl. Mol. Pathol..

[38] Mortimore G.E., Lardeux B.R., Adams C.E. (1988). Regulation of Microautophagy and Basal Protein Turnover in Rat Liver. Effects of Short-Term Starvation. J. Biol. Chem..

[39] Tuttle D.L., Lewin A.S., Dunn W.A. (1993). Selective Autophagy of Peroxisomes in Methylotrophic Yeasts. Eur. J. Cell Biol..

[40] Sakai Y., Koller A., Rangell L.K., Keller G.A., Subramani S. (1998). Peroxisome Degradation by Microautophagy in Pichia Pastoris: Identification of Specific Steps and Morphological Intermediates. J. Cell Biol..

[41] Campbell C.L., Thorsness P.E. (1998). Escape of Mitochondrial DNA to the Nucleus in Yme1 Yeast Is Mediated by Vacuolar-Dependent Turnover of Abnormal Mitochondrial Compartments. J. Cell Sci..

[42] Roberts P., Moshitch-Moshkovitz S., Kvam E., O’Toole E., Winey M., Goldfarb D.S. (2003). Piecemeal Microautophagy of Nucleus in Saccharomyces Cerevisiae. Mol. Biol. Cell.

[43] Van Zutphen T., Todde V., de Boer R., Kreim M., Hofbauer H.F., Wolinski H., Veenhuis M., van der Klei I.J., Kohlwein S.D. (2014). Lipid Droplet Autophagy in the Yeast Saccharomyces Cerevisiae. Mol. Biol. Cell.

[44] Schuck S., Gallagher C.M., Walter P. (2014). ER-Phagy Mediates Selective Degradation of Endoplasmic Reticulum Independently of the Core Autophagy Machinery. J. Cell Sci..

[45] Yang X., Zhang W., Wen X., Bulinski P.J., Chomchai D.A., Arines F.M., Liu Y.-Y., Sprenger S., Teis D., Klionsky D.J. (2020). TORC1 Regulates Vacuole Membrane Composition through Ubiquitin- and ESCRT-Dependent Microautophagy. J. Cell Biol..

[46] Schuck S. (2020). Microautophagy—Distinct Molecular Mechanisms Handle Cargoes of Many Sizes. J. Cell Sci..

[47] Mesquita A., Glenn J., Jenny A. (2021). Differential Activation of EMI by Distinct Forms of Cellular Stress. Autophagy.

[48] Uytterhoeven V., Lauwers E., Maes I., Miskiewicz K., Melo M.N., Swerts J., Kuenen S., Wittocx R., Corthout N., Marrink S.-J. (2015). Hsc70-4 Deforms Membranes to Promote Synaptic Protein Turnover by Endosomal Microautophagy. Neuron.

[49] Leung K.F., Dacks J.B., Field M.C. (2008). Evolution of the Multivesicular Body ESCRT Machinery; Retention Across the Eukaryotic Lineage. Traffic.

[50] Spang A., Saw J.H., Jørgensen S.L., Zaremba-Niedzwiedzka K., Martijn J., Lind A.E., van Eijk R., Schleper C., Guy L., Ettema T.J.G. (2015). Complex Archaea That Bridge the Gap between Prokaryotes and Eukaryotes. Nature.

[51] Hatano T., Palani S., Papatziamou D., Salzer R., Souza D.P., Tamarit D., Makwana M., Potter A., Haig A., Xu W. (2022). Asgard Archaea Shed Light on the Evolutionary Origins of the Eukaryotic Ubiquitin-ESCRT Machinery. Nat. Commun..

[52] Cuervo A.M., Dice J.F. (2000). Unique Properties of Lamp2a Compared to Other Lamp2 Isoforms. J. Cell Sci..

[53] Gough N.R., Hatem C.L., Fambrough D.M. (1995). The Family of LAMP-2 Proteins Arises by Alternative Splicing from a Single Gene: Characterization of the Avian LAMP-2 Gene and Identification of Mammalian Homologs of LAMP-2b and LAMP-2c. DNA Cell Biol..

[54] Hatem C.L., Gough N.R., Fambrough D.M. (1995). Multiple MRNAs Encode the Avian Lysosomal Membrane Protein LAMP-2, Resulting in Alternative Transmembrane and Cytoplasmic Domains. J. Cell Sci..

[55] Nishino I., Fu J., Tanji K., Yamada T., Shimojo S., Koori T., Mora M., Riggs J.E., Oh S.J., Koga Y. (2000). Primary LAMP-2 Deficiency Causes X-Linked Vacuolar Cardiomyopathy and Myopathy (Danon Disease). Nature.

[56] Chi C., Leonard A., Knight W.E., Beussman K.M., Zhao Y., Cao Y., Londono P., Aune E., Trembley M.A., Small E.M. (2019). LAMP-2B Regulates Human Cardiomyocyte Function by Mediating Autophagosome–Lysosome Fusion. Proc. Natl. Acad. Sci. USA.

[57] Fujiwara Y., Furuta A., Kikuchi H., Aizawa S., Hatanaka Y., Konya C., Uchida K., Yoshimura A., Tamai Y., Wada K. (2013). Discovery of a Novel Type of Autophagy Targeting RNA. Autophagy.

[58] Fujiwara Y., Kikuchi H., Aizawa S., Furuta A., Hatanaka Y., Konya C., Uchida K., Wada K., Kabuta T. (2013). Direct Uptake and Degradation of DNA by Lysosomes. Autophagy.

[59] Dehal P., Boore J.L. (2005). Two Rounds of Whole Genome Duplication in the Ancestral Vertebrate. PLoS Biol..

[60] Ravi V., Venkatesh B. (2018). The Divergent Genomes of Teleosts. Annu. Rev. Anim. Biosci..

[61] Pasquier J., Cabau C., Nguyen T., Jouanno E., Severac D., Braasch I., Journot L., Pontarotti P., Klopp C., Postlethwait J.H. (2016). Gene Evolution and Gene Expression after Whole Genome Duplication in Fish: The PhyloFish Database. BMC Genom..

[62] Terasawa K., Kato Y., Ikami Y., Sakamoto K., Ohtake K., Kusano S., Tomabechi Y., Kukimoto-Niino M., Shirouzu M., Guan J.-L. (2021). Direct Homophilic Interaction of LAMP2A with the Two-Domain Architecture Revealed by Site-Directed Photo-Crosslinks and Steric Hindrances in Mammalian Cells. Autophagy.

[63] Wilke S., Krausze J., Büssow K. (2012). Crystal Structure of the Conserved Domain of the DC Lysosomal Associated Membrane Protein: Implications for the Lysosomal Glycocalyx. BMC Biol..

[64] Rout A.K., Strub M.-P., Piszczek G., Tjandra N. (2014). Structure of Transmembrane Domain of Lysosome-Associated Membrane Protein Type 2a (LAMP-2A) Reveals Key Features for Substrate Specificity in Chaperone-Mediated Autophagy. J. Biol. Chem..

[65] Terasawa K., Tomabechi Y., Ikeda M., Ehara H., Kukimoto-Niino M., Wakiyama M., Podyma-Inoue K.A., Rajapakshe A.R., Watabe T., Shirouzu M. (2016). Lysosome-Associated Membrane Proteins-1 and -2 (LAMP-1 and LAMP-2) Assemble via Distinct Modes. Biochem. Biophys. Res. Commun..

[66] Ikami Y., Terasawa K., Sakamoto K., Ohtake K., Harada H., Watabe T., Yokoyama S., Hara-Yokoyama M. (2022). The Two-Domain Architecture of LAMP2A Regulates Its Interaction with Hsc70. Exp. Cell Res..

[67] Williams M.A., Fukuda M. (1990). Accumulation of Membrane Glycoproteins in Lysosomes Requires a Tyrosine Residue at a Particular Position in the Cytoplasmic Tail. J. Cell Biol..

[68] Guarnieri F.G., Arterburn L.M., Penno M.B., Cha Y., August J.T. (1993). The Motif Tyr-X-X-Hydrophobic Residue Mediates Lysosomal Membrane Targeting of Lysosome-Associated Membrane Protein 1. J. Biol. Chem..

[69] Hunziker W., Harter C., Matter K., Mellman I. (1991). Basolateral Sorting in MDCK Cells Requires a Distinct Cytoplasmic Domain Determinant. Cell.

[70] Harter C., Mellman I. (1992). Transport of the Lysosomal Membrane Glycoprotein Lgp120 (Lgp-A) to Lysosomes Does Not Require Appearance on the Plasma Membrane. J. Cell Biol..

[71] Russ W.P., Engelman D.M. (2000). The GxxxG Motif: A Framework for Transmembrane Helix-Helix Association. J. Mol. Biol..

[72] Jalali Z., Parvaz N. (2020). Molecular Evolution of Autophagy Rate-Limiting Factor LAMP2 in Placental Mammals. Gene.

[73] Jumper J., Evans R., Pritzel A., Green T., Figurnov M., Ronneberger O., Tunyasuvunakool K., Bates R., Žídek A., Potapenko A. (2021). Highly Accurate Protein Structure Prediction with AlphaFold. Nature.

[74] Varadi M., Anyango S., Deshpande M., Nair S., Natassia C., Yordanova G., Yuan D., Stroe O., Wood G., Laydon A. (2022). AlphaFold Protein Structure Database: Massively Expanding the Structural Coverage of Protein-Sequence Space with High-Accuracy Models. Nucleic Acids Res..

